# Huge trichobezoar presenting as abdominal mass and weight loss: Case report

**DOI:** 10.1016/j.ijscr.2019.02.033

**Published:** 2019-03-07

**Authors:** Ayad Ahmad Mohammed, Sardar Hassan Arif

**Affiliations:** University of Duhok, College of Medicine, Department of Surgery, Iraq

**Keywords:** Bezoar, Trichobezoar, Trichophagia, Trichotillomania

## Abstract

**Introduction:**

Trichobezoar is a condition in which hair is accumulated in the stomach forming a ball like mass. Human hair is resistant to digestion and peristalsis therefore over time it accumulates in the gastric folds. Trichophagia and trichotillomania is a psychiatric disorder in which the affected person has the tendency to pull her or his own hair and ingest it. The condition is almost exclusively seen in females.

**Case presentation:**

A 48-year-old lady presented for the last 6 months with epigastric pain, early satiety and weight loss. There are episodic attacks of vomiting. Abdominal examination showed a large, 30 cm × 15 cm, firm, oval shaped mass occupying the left hypochondrial region and extending below the umbilicus. The mass was mobile from side to side but not from up and down it has smooth surface. The mass was non pulsatile. The CT scan of the abdomen was taken and it showed a large oval mass with interspersed gas. Endoscopy showed a huge ball of hair occupying the whole gastric cavity and extending to the upper part of the duodenum. During laparotomy a huge hair mass extracted from the stomach.

**Conclusion:**

Trichobezoar is an extremely rare condition that may be encountered during the surgical practice, high index of suspicion is needed to diagnose the condition and endoscopy is still the gold standard method for investigation and diagnosis. The main treatment modality if the open surgery. All patients need psychiatric consultation and long term follow up.

## Highlights

• Trichobezoar is a very rare disorder which may lead to surgical emergency.
• In cases of acute abdomen pain, laparotomy is necessary.
• Psychiatric consultation is mandatory to prevent recurrence.

## 1 Introduction

Bezoars are indigestible foods, fibers, or certain materials that remains inside the alimentary tract of human and certain animals. This condition may be seen after gastric surgery such as partial gastrectomy or may be due to pica or in mentally abnormal persons. In most affected patients the condition is associated with underlying psychiatric disorder [1,2].

Trichobezoar is a condition in which hair is accumulated in the stomach forming a ball like mass, when this hair extends to the small bowel is called Rapunzel syndrome. Human hair is resistant to digestion and peristalsis therefore over time it accumulates in the gastric folds and form large hair ball. Trichophagia and trichotillomania, habitual hair pulling, is a psychiatric disorder in which the affected person has the tendency to pull her or his own hair and ingest it, it is considered as a part of the impulsive disorders [[3], [4], [5], [6]].

Patients may present with weight loss of failure to gain weight in children, upper abdominal pain, vomiting, anemia which is usually iron deficiency anemia and painless mass in the left upper quadrant of the abdomen, mechanical intestinal obstruction. This hair ball may cause ulceration of the gastric mucosa, bleeding and perforation. A case of pancreatitis due to trichobezoar had been reported. There may be associated abnormal behavioral disorders [1,3,[6], [7], [8], [9]].

The condition is almost exclusively seen in females and mostly occur at young ages but cases from pediatric age groups have been reported [7,10].

The diagnosis is done mostly by endoscopy and visualizing the hair occupying the gastric cavity and may extend beyond the stomach [2,3].

CT scan of the abdomen typically shows the gastric cavity occupied by a large well defined oval mass with interspersed gas, oral contrast typically sparse within the mass, it may show associated complications like intestinal obstruction and gastric perforation [3,8,11].

The work of this case report has been reported in line with the SCARE criteria [12].

### 1.1 Patient information

A 48-year-old lady presented for the last 6 months with dull aching epigastric pain, early satiety and weight loss, she has not recorded the degree of weight loss but she knows that from her clothes. There are episodic attacks of vomiting which non-bilious and not containing blood.

### 1.2 Clinical findings

**Fig. 1:**
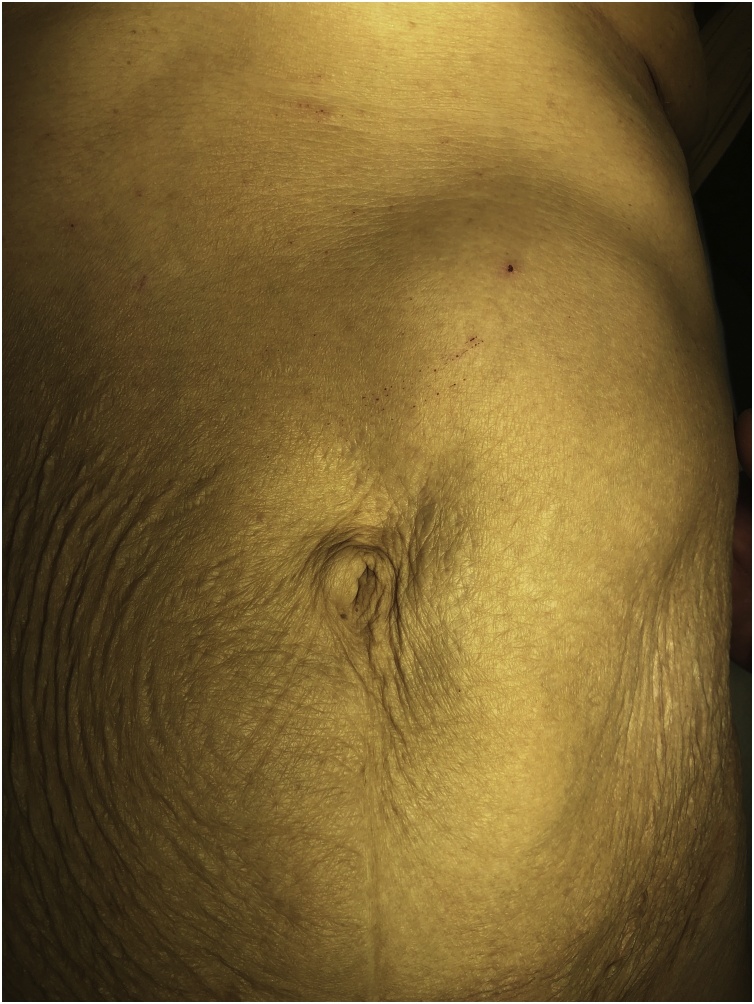
Picture of the abdomen of the patient showing the large visible mass in the left upper quadrant and extending below the umbilicus.

During examination the patient was thin, with no pallor or jaundice. The vital signs were normal. Abdominal examination showed a large, 30 cm × 15 cm, firm oval shaped mass occupying the left hypochondrial region and extending below the umbilicus. The mass was mobile from side to side but not from up and down it has smooth surface and was not attached to the abdominal wall. The mass was non pulsatile and we can feel between the mass and the costal margin. The bowel sounds were normal and there was no any abnormal bruit Fig. 1.

### 1.3 Diagnostic assessment

**Fig. 2:**
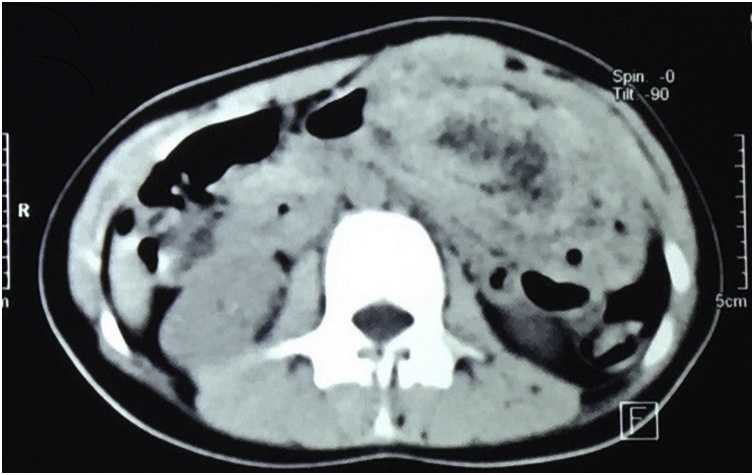
CT scan of the abdomen showing a large oval shaped intra-gastric mass with interspersed air.

The CT scan of the abdomen was taken and it showed a large oval mass occupying the gastric cavity with interspersed gas. Endoscopy showed a huge ball of hair occupying the whole gastric cavity and extending to the upper part of the duodenum Fig. 2.

### 1.4 Therapeutic intervention

**Fig. 3:**
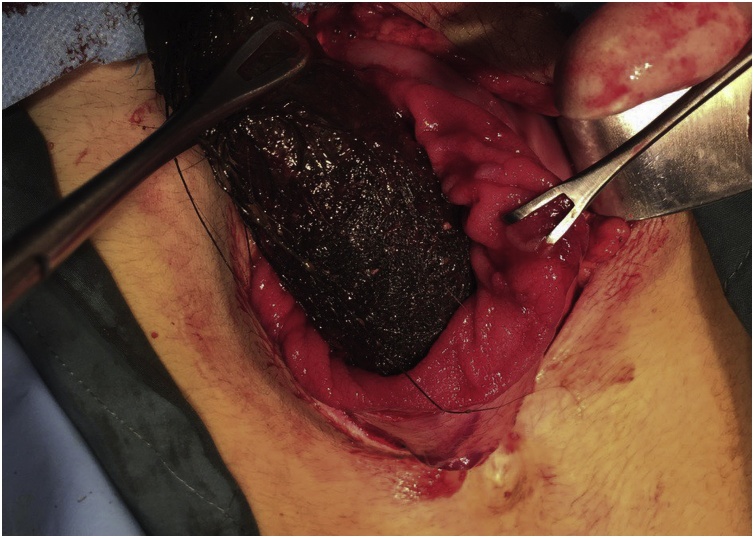
Intraoperative picture showing the hair ball being extracted from the stomach.

**Fig. 4:**
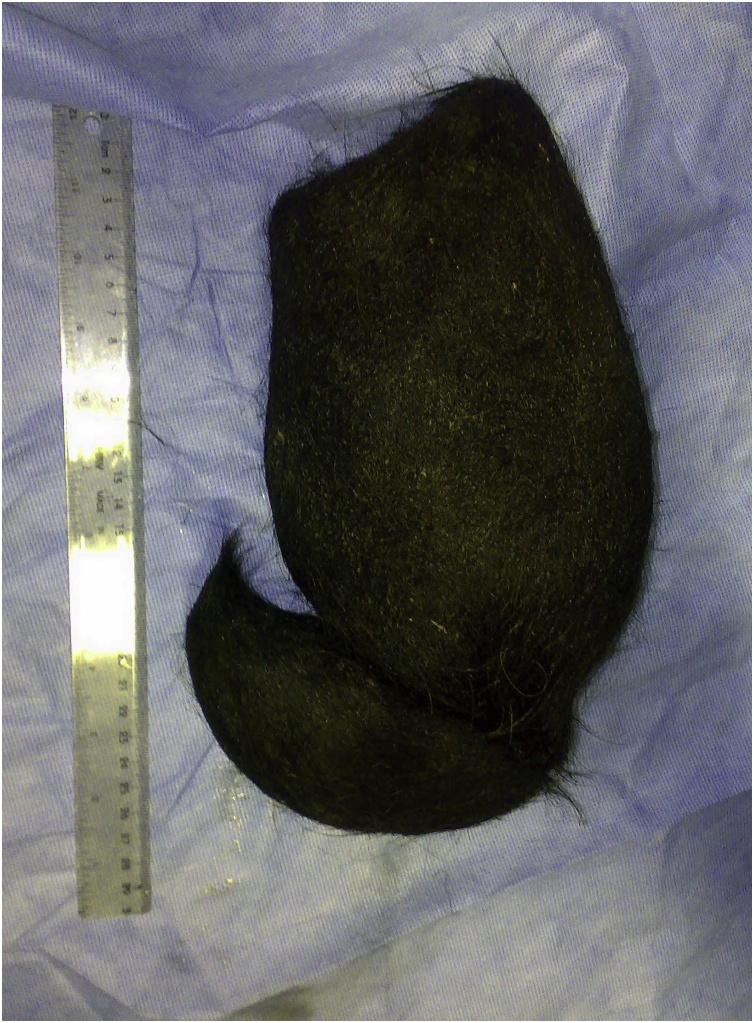
A huge hair ball after being extracted from the stomach.

During laparotomy with upper midline incision and longitudinal gastrostomy a huge mass formed from hair and filling the gastric cavity was extracted. Multiple associate ulcer in the gastric mucosa found, biopsy taken and the histopathological examination showed no malignancy. The stomach closed in 2 layers with inner continuous and outer interrupted suturing technique using 2/0 silk suture Fig. 3, Fig. 4.

### 1.5 Follow-up and outcomes

Nasogastric tube remains inside for 2 days and the oral intake started at the third day after surgery. The patient discharged home after 5 days in a good medical condition.

## 2 Discussion

Trichobezoar should be treated surgically to relieve the symptoms and to prevent complications. High index of suspicion is required to diagnose this condition [3].

Cases pf primary trichobezoar in the jejunum or the terminal ileum have been reported without any portion inside the stomach [10].

Most cases are treated with surgical extraction of the hair by the conventional open surgery. The surgery can be done using the hand assisted laparoscopic technique. Endoscopy usually fail to extract the hair unless if small in size, but successful extraction may be done when using mechanical and laser fragmentation of the hair [4,13].

Laparotomy is still the preferred method of treatment due to the very high success rate, shorter operation time, less complications and ability to examine the whole gastrointestinal tract for possibility of fragmentation and impaction of some pieces at the small bowel [4].

Trichobezoar is mostly associated with many psychiatric disorders such as trichotillomania, anxiety and depressive disorders, obsessive-compulsive neurosis, pica, anorexia nervosa, and body dysmorphic disorder. Most patients need psychiatric consultation and long term follow up after surgery to avoid recurrent bezoar formation although the recurrence rate is not estimated due to rarity of this condition [3,4,8].

### 2.1 Patient perspective

The patient denies at first any history of hair ingestion but after the endoscopy she said that she has history of hair ingestion for the last 5 years, after the surgery the patient was happy with the result of the operation and she accepted to have a psychiatric consultation.

## Conflicts of interest

The author has no conflicts of interest to declare.

## Sources of funding

None.

The authors are the source of the funding.

## Ethical approval

Ethical approval has been exempted by my institution for reporting this case.

## Consent

Written informed consent was obtained from the patient for publication of this case report and accompanying images.

## Author contribution

Dr Ayad Ahmad Mohammed and Dr Sardar Hassan Arif. contributed to the concept of reporting the case and the patient data recording.

Drafting the work, design, and revision done by Dr Ayad Ahmad Mohammed.

Dr Ayad Ahmad Mohammed took the consent from the patient for publishing the case.

Final approval of the work to be published was done by Dr Ayad Ahmad and Dr Sardar Hassan Arif.

## Registration of research studies

This work is case report and there is no need of registration.

## Guarantor

Dr Ayad Ahmad Mohammed is guarantor for the work.

## Provenance and peer review

Not commission, externally peer reviewed.

## References

[1] Phillips M.R., Zaheer S., Drugas G.T. (1998). Gastric trichobezoar: case report and literature review. Mayo Clin. Proc..

[2] Alsafwah S., Alzein M. (2000). Small bowel obstruction due to trichobezoar: role of upper endoscopy in diagnosis. Gastrointest. Endosc..

[3] Mohammed A.A., Arif S.H., Qadir R.H., Salih A.M., Kakamad F.H. (2018). Surgical extraction of a giant trichobezoar: a rare presentation. Int. J. Case Rep. Images.

[4] Gorter R., Kneepkens C., Mattens E., Aronson D., Heij H. (2010). Management of trichobezoar: case report and literature review. Pediatr. Surg. Int..

[5] Dalshaug G.B., Wainer S., Hollaar G.L. (1999). The Rapunzel syndrome (trichobezoar) causing atypical intussusception in a child: a case report. J. Pediatr. Surg..

[6] Lopes L.R., Oliveira P.S.P., Pracucho E.M., Camargo M.A., Neto C., de Souza J. (2010). The Rapunzel syndrome: an unusual trichobezoar presentation. Case Rep. Med..

[7] Deslypere J., Praet M., Verdonk G. (1982). An unusual case of the trichobezoar: the Rapunzel syndrome. Am. J. Gastroenterol..

[8] Pul N., Pul M. (1996). The Rapunzel syndrome (trichobezoar) causing gastric perforation in a child: a case report. Eur. J. Pediatr..

[9] Shawis R., Doig C. (1984). Gastric trichobezoar associated with transient pancreatitis. Arch. Dis. Child..

[10] Adhikari D.R., Vankipuram S., Tiwari A.R., Chaphekar A.P., Satardey R.S. (2015). Small intestinal obstruction secondary to jejunal trichobezoar removed per anum without an enterotomy: a case report. J. Clin. Diagn. Res. JCDR.

[11] O’sullivan M., McGreal G., Walsh J., Redmond H. (2001). Trichobezoar. J. R. Soc. Med..

[12] Agha R.A., Fowler A.J., Saeta A., Barai I., Rajmohan S., Orgill D.P. (2016). The SCARE statement: consensus-based surgical case report guidelines. Int. J. Surg..

[13] Palanivelu C., Rangarajan M., Senthilkumar R., Madankumar M. (2007). Trichobezoars in the stomach and ileum and their laparoscopy-assisted removal: a bizarre case. Singapore Med. J..

